# Complete Genome Sequence of GD487, a High-Virulence Strain of Human-Associated ST398 Methicillin-Susceptible Staphylococcus aureus

**DOI:** 10.1128/MRA.00686-19

**Published:** 2019-07-03

**Authors:** Jo-Ann McClure, Steven M. Shideler, Kunyan Zhang

**Affiliations:** a Centre for Antimicrobial Resistance, Alberta Health Services/Alberta Public Laboratories/University of Calgary, Calgary, Alberta, Canada; b Department of Pathology & Laboratory Medicine, University of Calgary, Calgary, Alberta, Canada; c Department of Microbiology, Immunology and Infectious Diseases, University of Calgary, Calgary, Alberta, Canada; d Department of Medicine, University of Calgary, Calgary, Alberta, Canada; e The Calvin, Phoebe and Joan Snyder Institute for Chronic Diseases, University of Calgary, Calgary, Alberta, Canada; Georgia Institute of Technology

## Abstract

Multilocus sequence type 398 (ST398) methicillin-susceptible Staphylococcus aureus (MSSA) has been shown to have augmented pathogenicity in humans. However, it has not been determined whether all ST398 strains are equally virulent. We present here the genome sequence of a high-virulence ST398 MSSA strain, GD487, to explore potential insights into ST398 virulence.

## ANNOUNCEMENT

Staphylococcus aureus multilocus sequence type 398 (ST398) was first reported as an animal pathogen affecting livestock and companion animals but was soon found associated with human diseases, even in the absence of animal contact (1–17). In humans, ST398 methicillin-susceptible S. aureus (MSSA) strains are commonly isolated and have been recognized as the causative agents in a number of severe infections in young healthy people, suggesting that MSSA strains have augmented pathogenicity in humans (2, 8, 18). Reports investigating the virulence of ST398 have focused on genetic factors of the group as a whole (10, 17, 19), with no specifics as to whether there are virulence differences within the lineage. The Caenorhabditis elegans infection model has proven to be a robust model for investigating S. aureus virulence (20, 21). We compared the virulence of ST398 strains in our collection using the model. Preliminary analysis found three lineages with high, moderate, or low virulence, with mean nematode killing rates of 90%, 67%, and 44%, respectively. Whole-genome sequencing was done to determine if there were specific factors present that could contribute to the noted levels of virulence. In separate reports, we presented the genomic sequences of a moderate-virulence (GD1108) and a low-virulence (GD1696) strain. Here, we present the complete chromosomal sequence of high-virulence strain GD487 to explore potential insights into ST398 sublineage virulence.

Strain GD487 was isolated from a school child from a prevalence survey in 2011 in Guangzhou, People’s Republic of China. Genomic DNA was isolated using phenol-chloroform extraction from a 37°C overnight culture of a single colony in brain heart infusion (BHI) broth. PacBio library preparation and DNA sequencing were performed at the Genome Quebec Innovation Centre in Montreal, Canada. A sheared large-insert library was generated using Covaris g-Tubes and the SMRTbell template prep kit 1.0 and then sequenced with PacBio RS II sequencing technology using one single-molecule real-time (SMRT) cell. Illumina library preparation and sequencing were performed at the Nicole Perkins Microbial Communities Core Laboratory at University of Calgary in Canada. The library was prepared using the Nextera XT library preparation kit using standard conditions, and then 600-cycle MiSeq v3 sequencing was done. Adapters were trimmed, and sequences with a quality score of <20 were removed from the raw Illumina reads with Cutadapt v1.15, and sequence quality was assessed with FastQC v0.11.5 (http://www.bioinformatics.babraham.ac.uk/projects/fastqc) (22). Hybrid genome assembly using the trimmed Illumina reads and filtered PacBio subreads (prepared by Genome Quebec) was done using the Unicycler v0.4.7 pipeline (SPAdes v3.13.0, minimap, Racon v1.3.2, and Pilon v.1.23), with GC content determined using QUAST v4.4 (23–28). Gene annotation was done using the NCBI Prokaryotic Genome Annotation Pipeline using the best-placed reference protein set (GeneMarkS-2+v4.8) (29). Default settings were used for each program.

Two contigs resulted from hybrid assembly of the GD487 reads, the chromosome and a plasmid (11, 006 bp). PacBio sequencing produced 98,884 raw reads covering 1,226,233,312 sequenced bases, with an average read length of 12,400 bp. Illumina sequencing produced 383,925 reads, with an average read length of 213 bp for R1 and 193 bp for R2. The estimated genome coverages were 395× and 30× for PacBio and Illumina reads, respectively, with a GC content of 32.96% for the assembled product. The resulting chromosome was 2,758,447 bp long, with 2,787 genes identified, of which 2,709 were coding sequences (CDS), 78 were RNA genes, and 87 were pseudogenes.

### Data availability.

The chromosomal genome sequence was deposited at GenBank under the accession number CP040229 and SRA accession numbers SRX5914559 (Illumina) and SRX5914560 (PacBio).
